# Ultrasound-controllable carbon monoxide nano-delivery systems for combined sonodynamic/gaseous therapies

**DOI:** 10.3389/fbioe.2025.1615481

**Published:** 2025-06-13

**Authors:** Chong Feng, Shuang Song, Xiaoyu Zhang, Jing Wang, Qingxin Meng, Tao Wang

**Affiliations:** ^1^ Ultrasound Department of Hong Qi Hospital of Mudanjiang Medical University, Mudanjiang, China; ^2^ Health Management Center, Mudanjiang First People’s Hospital, Mudanjiang, China; ^3^ Department of Biology, School of Basic Medical Sciences, Mudanjiang Medical University, Mudanjiang, China; ^4^ Ultrasound Department of the Second Affiliated Hospital of Mudanjiang Medical University, Mudanjiang, China

**Keywords:** sonodynamic therapy, gaseous therapy, breast cancer, hybrid materials, controlled release

## Abstract

**Indroduction:**

The integration of sonodynamic therapy (SDT) and carbon monoxide (CO) presents a promising synergistic strategy in cancer therapy owing to the unique advantage of CO in SDT sensitization. However, the development of SDT-compatible CO-delivery nanosystems remain a substantial challenge.

**Methods:**

Here, we developed an ultrastable and controllable CO nanoreservoir system through the integration of chlorine e6 (Ce6)-loaded, cancer cell membrane coating and iron carbonyl (Fe_3_CO_12_)-bridged mesoporous silica bodies (Fe_3_CO_12_-MSNs), which was specifically engineered to simultaneously achieve SDT and ultrasound (US)-responsive sustained CO release. Owing to the stabilization of Fe_3_CO_12_ within the silica framework, Fe_3_CO_12_-MSNs not only decreased unwanted CO leakage during transport but also enabled US-responsive matrix degradation accompanied by sustained CO release at tumor sites, which prolongs the therapeutic window of CO and maximizes the synergy of SDT and CO therapy.

**Results and Discussion:**

This nanoplatform-mediated combination therapies showed highly efficient antitumor effects and triggered a robust tumor-specific immune responses. When in combination with immune checkpoint blockers, the nanoplatform notably eradicate the breast cancer with low systematic toxicity. Overall, our work provides a promising nanoplatform with US-responsive and sustainable CO release for highly efficient and safe SDT/CO combined therapeis.

## 1 Introduction

Breast cancer has become one of the most common malignancies that severely threatens women’s health worldwide (Sun et al., 2017; Britt et al., 2020; Zhang et al., 2021). Traditional therapies, including surgery, radiotherapy, and chemotherapy, remain unsatisfactory because of their ineffectiveness and severe side effects (Castaneda and Strasser, 2017; Keilty et al., 2020; Zuo et al., 2024). Photodynamic therapy (PDT) and sonodynamic therapy (SDT), which use laser and ultrasound (US) to activate photosensitizers and sonosensitizers for the production of reactive oxygen species (ROS), have gained considerable attention owing to their facile, controllable, and noninvasive characteristics (Sun et al., 2023; Zhang et al., 2023). Compared to PDT, SDT has performed well in more types of cancer because of its higher therapeutic depth (Zhao et al., 2024). Although more and more evidence has proven the effectiveness of SDT, SDT is still unable to eliminate cancer cells. In addition to SDT, gaseous therapy, such as oxygen (O_2_), hydrogen (H_2_), carbon monoxide (CO), and nitric oxide (NO), has been developed as a supporting remedy for cancer treatments (Yu et al., 2018; Chen et al., 2019). Among gaseous molecules, CO, as a critical gasotransmitter that targets mitochondria and increases mitochondrial respiration, can sensitize tumor cells to ROS while protecting normal cells from oxidative stresses, thus considering a promising avenue to synergize with SDT (Zhao et al., 2019; Du et al., 2023). Unfortunately, direct inhalation of CO makes it difficult for CO to achieve the desired levels at tumor sites (Wang et al., 2019; Jin et al., 2021). Therefore, it is an urgent task to develop strategies for the efficient and controllable delivery of CO and sonosensitizers to maximize synergistic effects and minimize toxicity.

Metal carbonyl complexes (MCCs) are known as the most widely used CO-releasing molecules, which can reduce the side effects of direct inhalation (Wang et al., 2020; Zhang et al., 2024). Intensive efforts have been made to design various nanocarriers for preloading unstable MCCs to increase their accumulation at tumor sites. However, it remains challenging to prevent premature CO leakage during the transport of nanodrugs and achieve synchronous production of ROS and CO during the combination of SDT and gaseous therapy. Compared with traditional organic and inorganic materials, organic–inorganic hybrid materials are more promising as delivery vehicles by virtue of their integration of the stability of inorganic materials and controllability and biodegradability inherited from organic materials (Erigoni and Diaz, 2021; Liu et al., 2021; Wang et al., 2024). Among them, MCC-bridged mesoporous silica nanoparticles (MSNs), which lock unstable MCCs in a stable mesoporous silica framework, have performed well in decreasing unwanted MCC leakage and achieving ROS-responsive release (Lu et al., 2023). However, the potential of MCC-bridged MSNs in SDT has not yet been fully explored.

**SCHEME 1:**
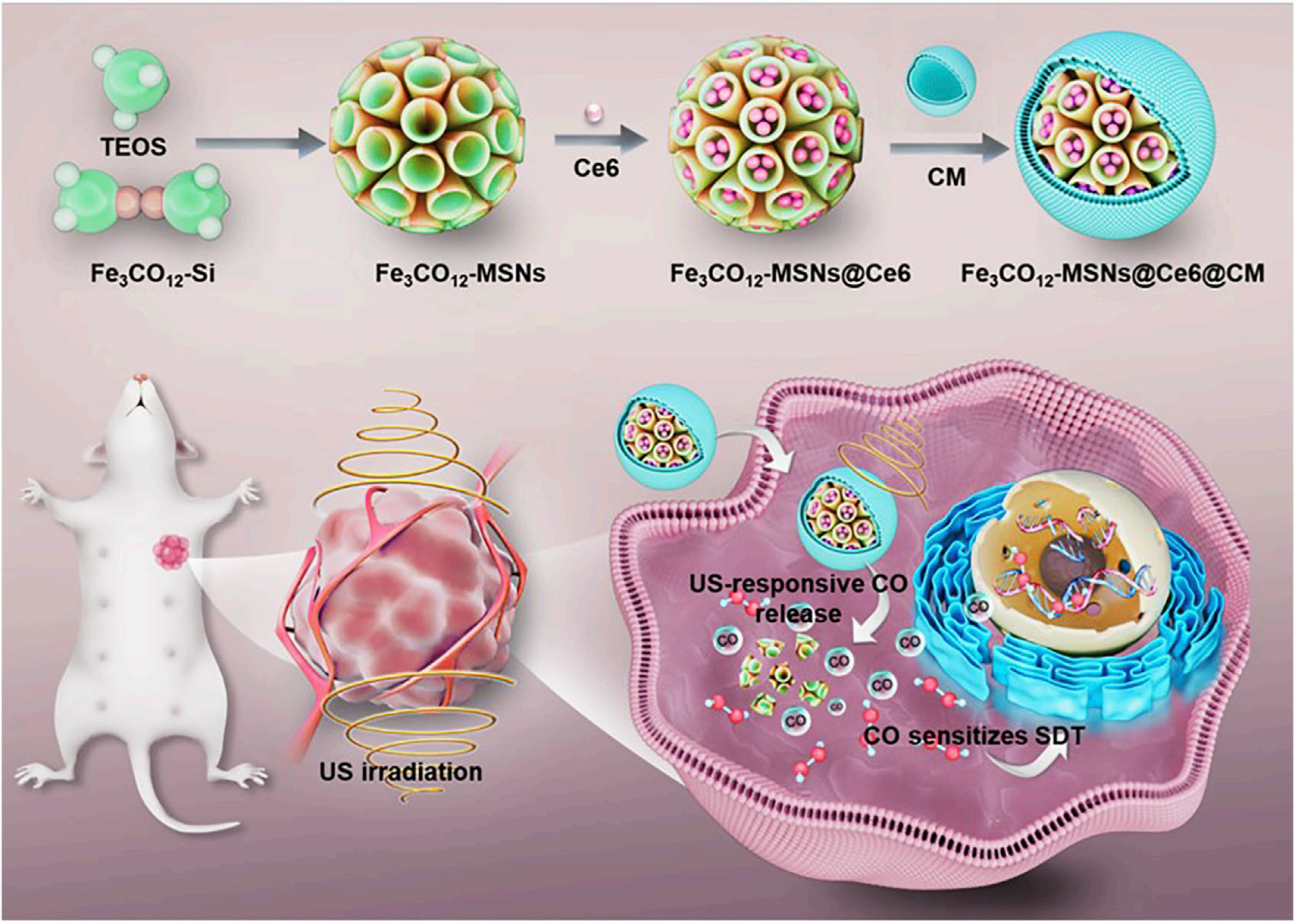
Schematic illustration of the fabrication of Fe_3_CO_12_-MSNs@Ce6@CM and its application for SDT/CO combination therapies of breast cancer.

In this study, we have fabricated an iron carbonyl (Fe_3_CO_12_)-bridged MSN (Fe_3_CO_12_-MSN) to preload sonosensitizers chlorine e6 (Ce6) for integrating SDT and CO gaseous therapy and then coated the cancer cell membrane to further improve the tumor target property. The prepared nanodrugs (Fe_3_CO_12_-MSNs@Ce6@CM) not only showed good stability in reducing CO leakage but also achieved biodegradation of the silica matrix and controllable release of CO in response to US irradiation. The sustainable release of CO triggered the maximal DNA damage to sensitize tumor cells to SDT and induce robust immunogenic cell death. Combined with immune checkpoint blockade therapy, the nanodrug enables the elimination of deeply metastatic tumors with low systemic toxicity. Our study provides a plausible strategy to integrate SDT and CO gaseous therapy for highly efficient and safe cancer treatments (Scheme 1).

## 2 Materials and methods

### 2.1 Preparation of Fe_3_CO_12_-MSNs

First, 0.25 g of Fe_3_(CO)_12_ was mixed with 0.353 g of 3-mercaptopropyltriethoxysilane (MPTES) in 50 mL of tetrahydrofuran (THF) and reacted at 70°C under nitrogen protection. After 2 h, the Fe_3_CO_12_-bridged organosilane (Fe_3_CO_12_-Si) was obtained by centrifugation and stored at − 20°C for subsequent use. To synthesize Fe_3_CO_12_-MSNs, 0.1 g of triethanolamine (TEAH3) and 0.3 g of CTAB were added to 20 mL of deionized water and heated at 80°C for 30 min. Then, 0.2 g tetraethyl orthosilicate (TEOS) and Fe_3_CO_12_-bridged organosilane were added into the mixture as silica precursors and reacted for another 4 h. Finally, Fe_3_CO_12_-MSNs were obtained by centrifugation and stored at 4°C for subsequent experiments.

### 2.2 Cell membrane coating

4T1 cells were resuspended in 20 mL of hypotonic lysis buffer and subjected to sequential centrifugation. The obtained cell ghosts were then resuspended in 2 mL of water and sonicated for 10 min. To obtain cell membrane vesicles, the resulting ghosts were serially extruded through polycarbonate membranes. To coat the cell membranes onto Fe_3_CO_12_-MSNs@Ce6, Fe_3_CO_12_-MSNs@Ce6 was mixed with cell membrane vesicles in deionized water and extruded through 200-nm polycarbonate membranes. The prepared Fe_3_CO_12_-MSNs@Ce6@CM was lyophilized and stored at 4°C for subsequent experiments.

### 2.3 Biodegradation and CO release

To investigate the degradation of Fe_3_CO_12_-MSNs@Ce6@CM, Fe_3_CO_12_-MSNs@Ce6@CM and MSNs@ Fe_3_CO_12_/Ce6@CM were dispersed in PBS solution with 0 or 100 μM of H_2_O_2_ under mild stirring. After 6 h, the X-ray US groups were exposed to US (40 kHz, 3.0 W/cm^2^, 50% duty cycle) for 5 min. Then, the samples were collected on days 0, 1, and 3 for transmission electron microscopy (TEM, JEOL, Ltd., Japan), and the concentration of Si in the supernatant was measured using ICP-OES at predetermined time points.

We used the hemoglobin (Hb) assay to measure the CO release of Fe_3_CO_12_-MSNs@Ce6@CM in response to H_2_O_2_ and US irradiation. In brief, 20 μM of Hb was dissolved in the PBS solution with 0 or 100 μM of H_2_O_2_ under nitrogen protection in the presence of sodium dithionite. Then, Fe_3_CO_12_-MSNs@Ce6@CM and MSNs@ Fe_3_CO_12_/Ce6@CM were added to the mixture solution. The mixture solution was irradiated by US for 5 min at 6 h. Next, the samples were collected at predetermined time points, and the absorbances of the samples were measured at 410 and 430 nm. The CO release was calculated using the following equation: concentration of CO = (I_410nm_ × 528.6 − I_430 nm_ × 304)/(I_410nm_ × 216.5 − I_430 nm_ × 442.4), where I_430nm_ and I_410nm_ represent the absorbance of the sample at wavelengths of 430 and 410 nm, respectively.

### 2.4 Cellular uptake and cytotoxicity

To assess the cellular uptake of Fe_3_CO_12_-MSNs@Ce6@CM, FITC-labeled Fe_3_CO_12_-MSNs@Ce6, MSNs@Fe_3_CO_12_/Ce6@CM, and Fe_3_CO_12_-MSNs@Ce6@CM were incubated with 4T1 cells for 4 h, respectively. Subsequently, the cells were fixed with paraformaldehyde for 10 min, stained with DAPI for 10 min, and observed using the confocal laser scanning microscope (CLSM; Olympus FV1000; Olympus, Tokyo, Japan). To quantify the cellular uptake, the cells were resuspended and detected using flow cytometry (BD Biosciences, Franklin Lakes, NJ, United States).

To investigate the cytotoxicity, 4T1 cells were planted onto 96-well plates at a density of 4 × 10^3^ cells/wells. After incubation overnight, Fe_3_CO_12_-MSNs@CM, Fe_3_CO_12_-MSNs@Ce6@CM, and MSNs@Fe_3_CO_12_/Ce6@CM were added into each well at various concentrations and incubated for 24 h. For the US treatment groups, 4T1 cells were exposed to US (40 kHz, 3.0 W/cm^2^, 50% duty cycle) for 5 min at 6 h post-administration. Then, cell viability was analyzed using CCK-8 assays.

### 2.5 Biodistribution

Animal experiments were approved by the Ethics Committee for the Use of Experimental Animals of Harbin Medical University and in accordance with the National Institute of Health Guide for the Care and Use of Laboratory Animals. To establish 4T1 tumor murine models, 1 mL of 4T1 cell suspension (5 × 10^6^) was injected into the mammary fat pads of female Balb/c mice. When the tumor volume reached approximately 800 mm^3^, 4T1 tumor-bearing mice were intravenously injected with Cy5.5-labeled Fe_3_CO_12_-MSNs@Ce6 and Fe_3_CO_12_-MSNs@Ce6@CM at a dose of 10 mg/kg. Then, major organs and tumors were harvested and weighed at different time points after administration and subsequently homogenized to measure fluorescence intensity.

### 2.6 Therapeutic effect *in vivo*

All the tumor models were randomized into seven groups and then intravenously injected with saline, Fe_3_CO_12_-MSNs@Ce6@CM (10 mg/kg), Fe_3_CO_12_-MSNs@Ce6@CM (10 mg/kg) plus αPD-L1 (1 mg/kg), and MSNs@Fe_3_CO_12_/Ce6@CM (10 mg/kg) plus αPD-L1 (1 mg/kg) in the absence or presence of US. Fe_3_CO_12_-MSNs@Ce6@CM or MSNs@Fe_3_CO_12_/Ce6@CM were administered every 3 days, and the tumor sites were irradiated with 1 MHz US at 1 W/cm^2^ for 1 min at 8 h post-injection. For the αPD-L1 treatment groups, αPD-L1 was intravenously injected on day 6. Tumor volumes were measured every 3 days using a digital caliper and calculated using the formula: tumor volume = length × width^2^ × 0.52. All the mice were euthanized on day 23, and the tumors were harvested and weighed.

### 2.7 Systemic toxicity evaluation

4T1 tumor-bearing mice were intravenously injected with Fe_3_CO_12_-MSNs@Ce6@CM (10 mg/kg) and Fe_3_CO_12_-MSNs@Ce6@CM (10 mg/kg) plus αPD-L1 (1 mg/kg) and exposed to 1 Hz US at 1 W/cm^2^ for 1 min. All the mice were euthanized on day 21. The major organs, including the liver, spleen, kidneys, heart, and lungs, were harvested, fixed, and stained with hematoxylin–eosin (H&E). Blood was collected, and the levels of aspartate aminotransferase (AST), alanine aminotransferase (ALT), alkaline phosphatase (ALP), and blood urea nitrogen (BUN) were detected using ELISA kits.

### 2.8 Statistics

GraphPad Prism was used for statistical analysis, with Student’s t-test applied for comparing two groups, while one-way ANOVA (Tukey’s multiple comparison test) or two-way ANOVA (Tukey’s and Sidak’s multiple comparisons test) was used for analyzing differences among multiple groups. Data are presented as the mean ± SD, with significance levels denoted as *P < 0.05, **P < 0.01, and ***P < 0.001, where P < 0.05 indicates statistical significance.

## 3 Results and discussion

**FIGURE 1:**
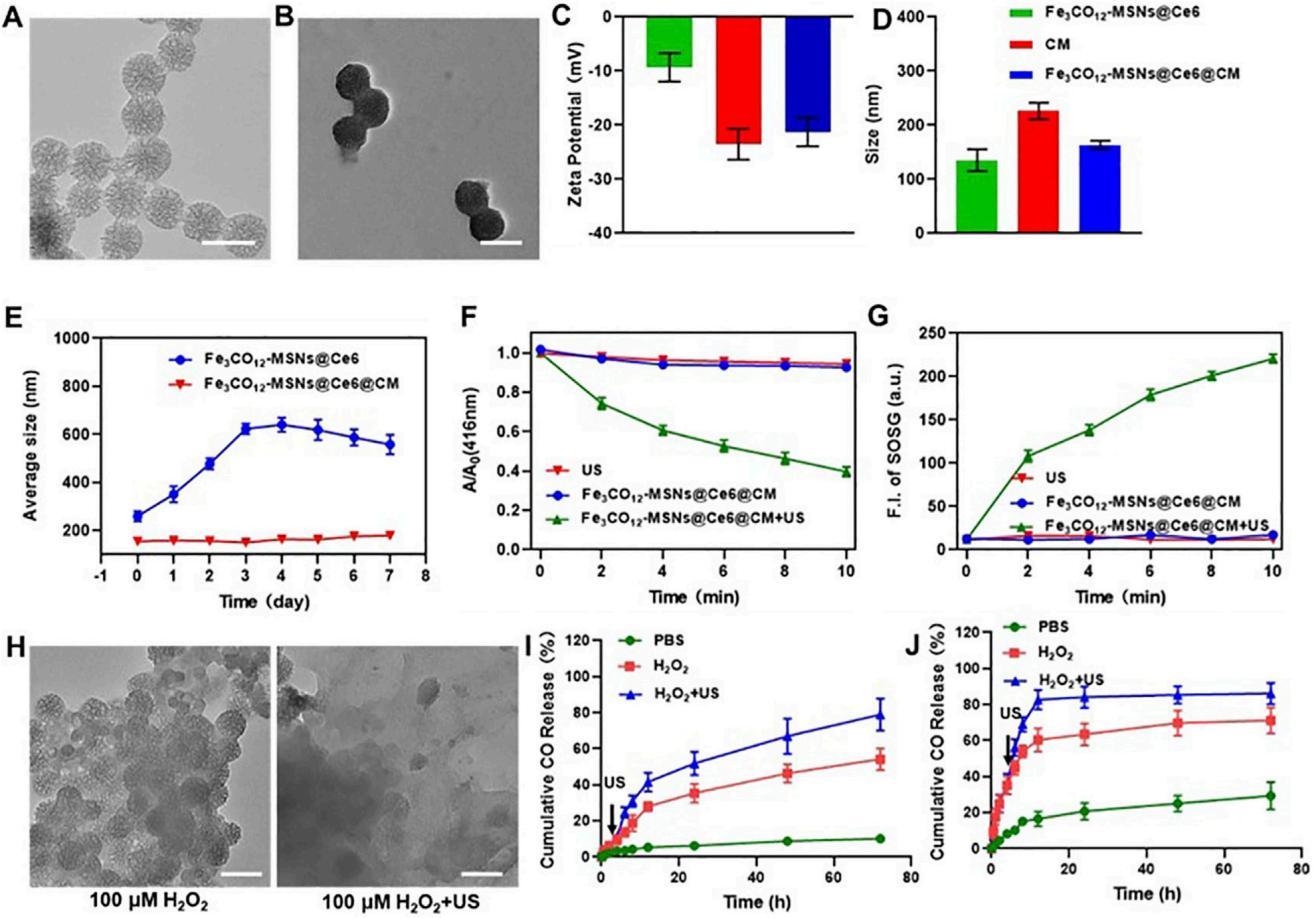
Characterization, biodegradation, and CO release of Fe_3_CO_12_-MSNs@Ce6@CM. **(A)** TEM image of Fe_3_CO_12_-MSNs; scale bar, 100 nm. **(B)** TEM image of Fe_3_CO_12_-MSNs@Ce6@CM; scale bar, 100 nm. **(C)** Surface zeta potential of Fe_3_CO_12_-MSNs@Ce6, CM vesicles, and Fe_3_CO_12_-MSNs@Ce6@CM; n = 3. **(D)** Size distribution of Fe_3_CO_12_-MSNs@Ce6, CM vesicles, and Fe_3_CO_12_-MSNs@Ce6@CM; n = 3. **(E)** Averaged size of Fe_3_CO_12_-MSNs@Ce6 and Fe_3_CO_12_-MSNs@Ce6@CM after storage in the cell medium; n = 3. **(F)** Comparison of the SDT effect of Fe_3_CO_12_-MSNs@Ce6@CM and MSNs@Fe_3_O_4_/Ce6@CM using a DPBF probe; n = 3. **(G)** Singlet oxygen generation of Fe_3_CO_12_-MSNs@Ce6@CM and MSNs@Fe_3_O_4_/Ce6@CM with US irradiation. **(H)** Morphology of Fe_3_CO_12_-MSNs in the presence of 100 μM H_2_O_2_ solution with or without US irradiation; scale bar, 100 nm. **(I, J)** Cumulative CO release from Fe_3_CO_12_-MSNs@Ce6@CM **(I)** and MSNs@Fe_3_O_4_/Ce6@CM **(J)** in the presence of H_2_O_2_ and US irradiation; n = 3. Data are presented as the mean ± SD.

We prepared Fe_3_CO_12_-MSNs using a sol–gel method, with tetraethyl orthosilicate (TEOS) and Fe_3_CO_12_-bridged organosilane as the precursors and cetyltrimethylammonium bromide (CTAB) as a structure-directing agent. As shown in Figure 1A, Fe_3_CO_12_-MSNs exhibited a spherical shape with a diameter of ∼100 nm. N_2_ adsorption–desorption isotherms indicated that Fe_3_CO_12_-MSNs had a pore volume of 1.02 cm^3^/g, a larger surface area of 613.5 m^2^/g, and uniform pore size distribution of 3.6 nm, which suggested the excellent drug-loading capability of Fe_3_CO_12_-MSNs (Supplementary Figure S1). The drug-loading content of Fe_3_CO_12_-MSNs@Ce6 was calculated to be 9.7%. To improve the colloidal stability and tumor targeting, we coated murine breast cancer 4T1 cell membranes onto the surface of Fe_3_CO_12_-MSNs. The formed Fe_3_CO_12_-MSNs@Ce6@CM showed an obvious core-shell structure with a thin lipid shell and a slightly larger hydrodynamic size than that of Fe_3_CO_12_-MSNs@Ce6 (Figures 1B,C). Furthermore, the surface potential of Fe_3_CO_12_-MSNs@Ce6@CM was more negative than Fe_3_CO_12_-MSNs@Ce6, which was approximate to that of cell membranes (Figure 1D). These results confirmed the successful coating of cell membranes onto Fe_3_CO_12_-MSNs@Ce6. Additionally, Fe_3_CO_12_-MSNs@Ce6@CM displayed good monodispersity after storage in cell medium for 24 h, whereas aggregation appeared in Fe_3_CO_12_-MSNs@Ce6 in the cell medium, indicating that the coating of cell membranes improved the colloidal stability of Fe_3_CO_12_-MSNs@Ce6 (Figure 1E). To explore the potential of Fe_3_CO_12_-MSNs@Ce6@CM as sonosensitizers, we detected the ROS generation of Fe_3_CO_12_-MSNs@Ce6@CM under irradiation using a 1,3-diphenylisobenzofuran (DPBF) probe. As shown in Figure 1F, Fe_3_CO_12_-MSNs@Ce6@CM did not produce ROS in the absence of US. However, the generation of ROS was detected when Fe_3_CO_12_-MSNs@Ce6@CM was irradiated by US. Moreover, the amount of ROS was increased with the extension of US irradiation. Consistent results regarding the generation of singlet oxygen (^1^O_2_) were measured using a singlet oxygen sensor green (SOSG) probe. These results indicated the potential of Fe_3_CO_12_-MSNs@Ce6 to function as effective sonosensitizers for cancer SDT.

We next investigated the degradation and release behavior of Fe_3_CO_12_-MSNs@Ce6@CM in response to US irradiation. Fe_3_CO_12_-MSNs collapsed into irregular aggregates in the PBS solution containing 100 μΜ H_2_O_2_, simulating a tumor microenvironment, but were unable to further disassemble (Figure 1G). In contrast, Fe_3_CO_12_-MSNs completely degrade in 100 μΜ H_2_O_2_ with US irradiation. This phenomenon could be explained by the cleavage of the Fe–CO bond by the strong oxidative H_2_O_2_, while the generation of ROS by US inside the MSNs directly breaks the Fe–CO bond to promote degradation. The ROS- and US-dual-responsive degradation property might contribute to improving the specificity of CO delivery in tumors. We subsequently explored the CO release from Fe_3_CO_12_-MSNs@Ce6@CM in PBS or 100 μΜ H_2_O_2_ with or without US. To further verify the advantages of Fe_3_CO_12_-MSNs@Ce6@CM in CO release, inorganic MSNs were prepared to load Ce6 and Fe_3_CO_12_ and coated with 4T1 cell membranes (MSNs@Fe_3_CO_12_/Ce6@CM) as a comparison. The increased hydrodynamic size and negative surface potential of MSNs@ Fe_3_CO_12_/Ce6@CM indicated the successful coating of CM onto MSNs@Fe_3_CO_12_/Ce6 (Supplementary Figure S2). Additionally, the coating of CM improved the colloidal stability of MSNs@Fe_3_CO_12_/Ce6@CM (Supplementary Figure S3). Notably, MSNs showed a similar morphology to Fe_3_CO_12_-MSN but could not degrade in 100 μΜ H_2_O_2_ solution or under US irradiation (Supplementary Figure S4). Furthermore, both Fe_3_CO_12_-MSNs@Ce6@CM and MSNs@ Fe_3_CO_12_/Ce6@CM showed ROS- and US-dual-responsive CO release behavior. However, CO release from Fe_3_CO_12_-MSNs@Ce6@CM was more sustained in the presence of H_2_O_2_ and H_2_O_2_ plus US than that from MSNs@Fe_3_CO_12_/Ce6@CM under the same stimulus. The sustainable CO release behavior may help maintain the therapeutic concentration of CO in tumors for prolonging its therapeutic window since CO has high diffusivity and poor solubility, which may address the challenge of therapeutic gases failing to achieve prolonged high-concentration enrichment at target sites. On the other hand, the leakage of CO from MSNs@Fe_3_CO_12_/Ce6@CM in the PBS solution reached 29.3% without 100 μΜ H_2_O_2_ or US irradiation, whereas only 10.1% CO was released from Fe_3_CO_12_-MSNs@Ce6@CM after 24 h. These results suggested that Fe_3_CO_12_-MSNs@Ce6@CM were much more stable than MSNs@Fe_3_CO_12_/Ce6@CM, which helped decrease unwanted CO leakage during circulation.

**FIGURE 2:**
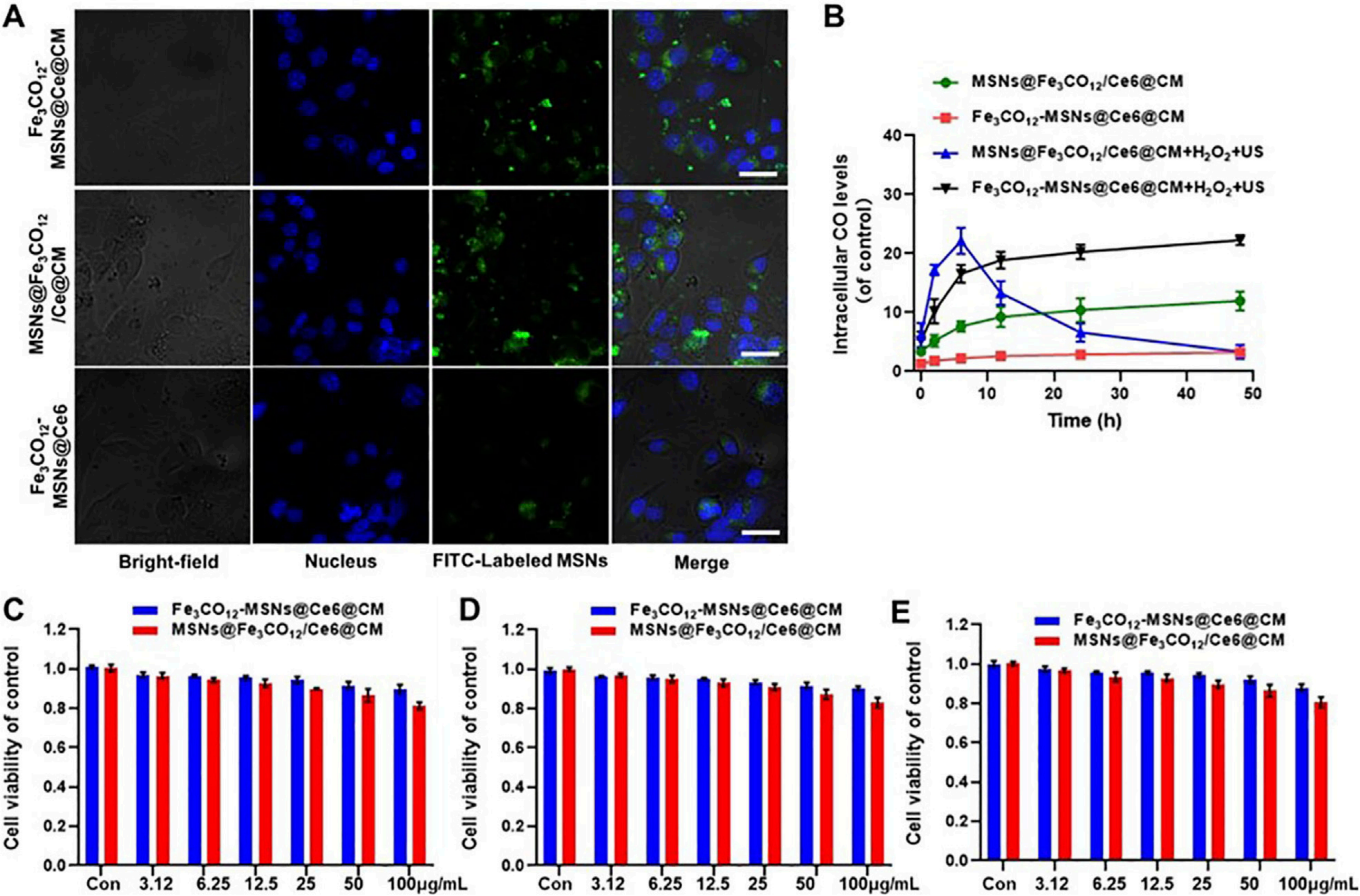
Cellular uptake and cytotoxicity of Fe_3_CO_12_-MSNs@Ce6@CM. **(A)** CLSM images of 4T1 cells after incubation with Fe_3_CO_12_-MSNs@Ce6, Fe_3_CO_12_-MSNs@Ce6@CM, and MSNs@Fe_3_CO_12_/Ce6@CM for 4 h; scale bar, 10 μm. **(B)** Intracellular CO level after treatment with Fe_3_CO_12_-MSNs@Ce6@CM and MSNs@Fe_3_CO_12_/Ce6@CM with or without 100 μM H_2_O_2_ plus US irradiation; n = 3. **(C–F)** Cytotoxicity of Fe_3_CO_12_-MSNs@Ce6@CM and MSNs@Fe_3_CO_12_/Ce6@CM against 4T1 cells **(C)**, MCF-7 cells **(D)**, and HUVECs **(E)**; n = 4. Data are presented as the mean ± SD.

Encouraged by the US-activated ROS generation and US-controllable CO release, we sought to investigate the cytotoxicity of Fe_3_CO_12_-MSNs@Ce6@CM *in vitro*. Endocytosis plays a vital role in cytotoxicity. Therefore, we first investigated the cellular uptake of Fe_3_CO_12_-MSNs@Ce6, Fe_3_CO_12_-MSNs@Ce6@CM, and MSNs@Fe_3_CO_12_/Ce6@CM in 4T1 cells. All the nanoparticles could be taken up by 4T1 cells, and Fe_3_CO_12_-MSNs@Ce6@CM exhibited a similar cellular uptake to MSNs@Fe_3_CO_12_/Ce6@CM (Figure 2A; Supplementary Figure S5). Additionally, Fe_3_CO_12_-MSNs@Ce6@CM showed higher cellular internalization efficiency than MSNs@Fe_3_CO_12_/Ce6, which was attributed to the coating of cancer cell membranes. Then, we investigated the intracellular CO delivery using the FL-CO-1 probe, which could generate 480 nm fluorescence after binding with CO. As shown in Figure 2B, lower fluorescent intensity was detected in Fe_3_CO_12_-MSNs@Ce6@CM than in MSNs@Fe_3_CO_12_/Ce6@CM without the stimulus of H_2_O_2_ or US, indicating that Fe_3_CO_12_-MSNs@Ce6@CM had better stability than MSNs@Fe_3_CO_12_/Ce6@CM. Notably, intracellular CO from Fe_3_CO_12_-MSNs@Ce6@CM showed a more sustained presence than that from MSNs@Fe_3_CO_12_/Ce6@CM in the presence of H_2_O_2_ and US, owing to the sustainable CO release of Fe_3_CO_12_-MSNs@Ce6@CM, which might contribute to enhancing the efficacy of CO therapy in tumors when US irradiation is applied. Then, we measured the cytotoxicity of Fe_3_CO_12_-MSNs@Ce6@CM toward 4T1 cells, MCF-7 cells, and HUVECs after 24 h using CCK-8 assays. As shown in Figures 2C–E and Supplementary Figure S6, MSNs@Fe_3_CO_12_/Ce6@CM showed slightly higher cytotoxicity than Fe_3_CO_12_-MSNs@Ce6@CM without US irradiation after 24 h and 72 h of incubation, likely due to the easier leakage of CO. The lower cytotoxicity of Fe_3_CO_12_-MSNs@Ce6@CM suggested that it had better biomedical application prospects.

**FIGURE 3:**
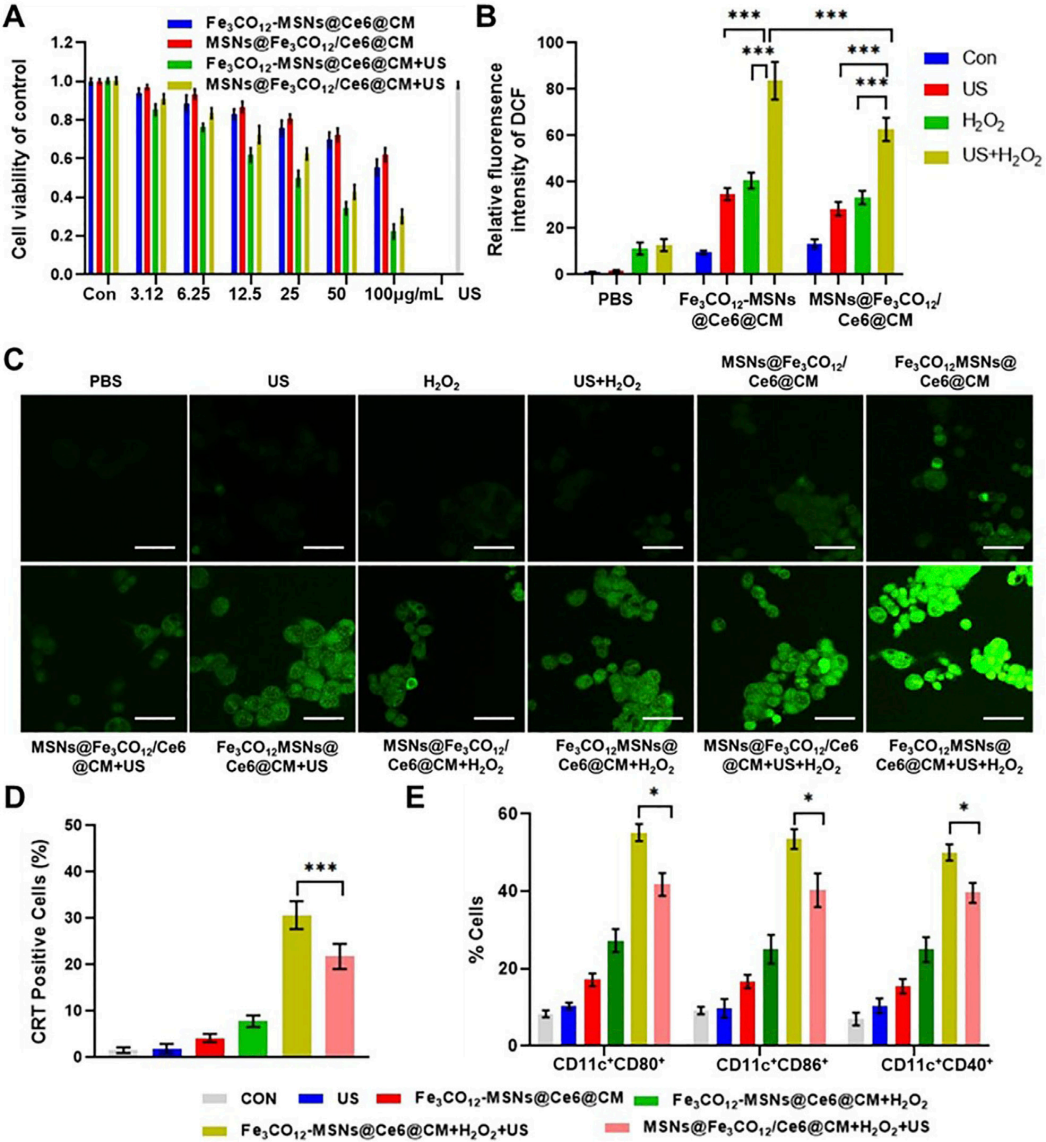
*In vitro* combined therapeutic effects of Fe_3_CO_12_-MSNs@Ce6@CM. **(A)** Cell viability of 4T1 cells after treatments with Fe_3_CO_12_-MSNs@Ce6@CM and MSNs@Fe_3_CO_12_/Ce6@CM in the presence of 100 μM H_2_O_2_ with or without US irradiation; n = 3. **(B)** Quantitative analysis of ROS fluorescence intensity for various treatments; n = 3. **(C)** Fluorescence images of ROS in 4T1 cells after various treatment; scale bar, 20 μm. **(D)** Percentage of CRT-positive cells after various treatments; n = 3. **(E)** Measurement of DC maturation markers CD80, CD86, and CD40 after incubation with differently treated 4T1 cells for 24 h; n = 4. Data are presented as the mean ± SD. *p < 0.05, **p < 0.01, and ***p < 0.001.

To further explore the therapeutic effect of Fe_3_CO_12_-MSNs@Ce6@CM *in vitro*, 4T1 cells were incubated with various concentrations of Fe_3_CO_12_-MSNs@Ce6@CM, Fe_3_CO_12_-MSNs@Ce6@CM, and MSNs@Fe_3_CO_12_/Ce6@CM with the addition of 100 μM, simulating a tumor microenvironment in the absence or presence of US irradiation. As shown in Figure 3A, both Fe_3_CO_12_-MSNs@Ce6@CM and MSNs@Fe_3_CO_12_/Ce6@CM showed concentration-dependent toxicity toward 4T1 cells. When exposed to US irradiation, the therapeutic effects of Fe_3_CO_12_-MSNs@Ce6@CM and MSNs@Fe_3_CO_12_/Ce6@CM were obviously enhanced, indicating their CO and SDT combination therapies. Notably, Fe_3_CO_12_-MSNs@Ce6@CM induced more 4T1 cell death than MSNs@Fe_3_CO_12_/Ce6@CM in the presence of 100 μΜ H_2_O_2_ and US irradiation, owing to the sustained CO release of Fe_3_CO_12_-MSNs@Ce6@CM. For further validation, we measured the intracellular ROS level after various treatments using 2ʹ,7ʹ-dichlorofluorescein diacetate (DCFH-DA), a nonfluorescent probe that can react with intracellular ROS to form fluorescent 2ʹ,7ʹ-dichlorofluorescein (DCF). As shown in Figures 3B,C, more intracellular ROS was detected in the cells after the treatment with MSNs@Fe_3_CO_12_/Ce6@CM than that with Fe_3_CO_12_-MSNs@Ce6@CM in the absence of US and H_2_O_2_, possibly due to the more leakage of CO from MSNs@Fe_3_CO_12_/Ce6@CM. Additionally, either US or H_2_O_2_ could increase intracellular ROS generation of MSNs@Fe_3_CO_12_/Ce6@CM and Fe_3_CO_12_-MSNs@Ce6@CM, and the combination of US and H_2_O_2_ induced more ROS generation of MSNs@Fe_3_CO_12_/Ce6@CM and Fe_3_CO_12_-MSNs@Ce6@CM. Furthermore, Fe_3_CO_12_-MSNs@Ce6@CM led to the highest ROS compared to MSNs@Fe_3_CO_12_/Ce6@CM under H_2_O_2_ and US stimulation. These results indicated that Fe_3_CO_12_-MSNs@Ce6@CM had great potential in sensitizing SDT, owing to its sustained CO release property. Considering that SDT could induce immunogenic cell death (ICD) to promote antitumor immune responses, we detected the ability of Fe_3_CO_12_-MSNs@Ce6@CM to induce ICD effects by measuring calreticulin (CRT) exposure and the secretion of chromatin-binding protein high-mobility group B1 (HMGB1). As expected, Fe_3_CO_12_-MSNs@Ce6@CM triggers more CRT-positive cells and higher release of HMGB1 than MSNs@Fe_3_CO_12_/Ce6@CM in the presence of US and H_2_O_2_ (Figure 3D; Supplementary Figure S7). To further evaluate the immunological effects of Fe_3_CO_12_-MSNs@Ce6@CM-mediated combination therapies, we incubated 4T1 cells after various treatments with dendritic cells (DCs). We found that Fe_3_CO_12_-MSNs@Ce6@CM plus US-treated 4T1 cells induced the most DC maturation (Figure 3E). These results indicated that Fe_3_CO_12_-MSNs@Ce6@CM-mediated combination therapies could activate a strong antitumor immune response.

**FIGURE 4:**
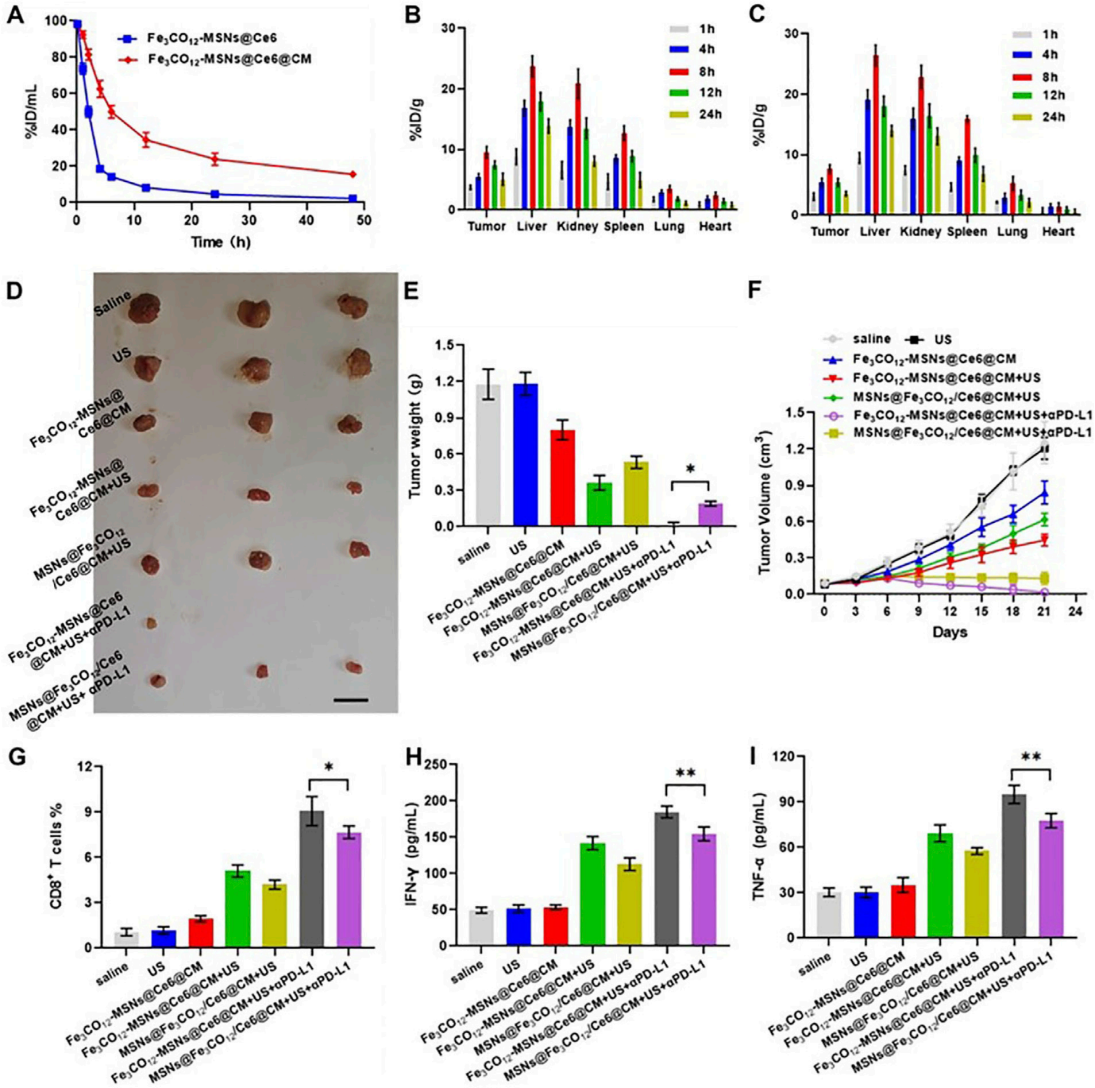
*In vivo* combination therapeutic effects. **(A)** Blood circulation of Fe_3_CO_12_-MSNs@Ce6@CM and Fe_3_CO_12_-MSNs@Ce6 in mice, n = 3. **(B,C)** Biodistribution of Fe_3_CO_12_-MSNs@Ce6@CM **(B)** and Fe_3_CO_12_-MSNs@Ce6 **(C)** after intravenous injection to 4T1 tumor-bearing mice; n = 3. **(D)** Tumor photographs of mice after various treatments, the scale bar =1 cm. **(E)** Tumor weights of mice after various treatments, n = 3. **(F)** Tumor volumes, n = 3. **(G)** Quantitative analysis of CD8+T cells in tumors of after various treatments on day 8, n = 3. **(H,I)** Secretion of IFN-γ **(H)** and TNF-α **(I)** after various treatments, n=3. Data are presented as the mean ± SD, *p < 0.05, **p < 0.01, ***p < 0.001.

After demonstrating the anticancer effect and ICD induction of Fe_3_CO_12_-MSNs@Ce6@CM *in vitro*, we explored its therapeutic effect *in vivo*. Owing to the coating of cancer cell membranes, Fe_3_CO_12_-MSNs@Ce6@CM exhibited prolonged blood circulation and higher tumor accumulation efficiency than Fe_3_CO_12_-MSNs@Ce6 (Figures 4A–C). The tumor accumulation of Fe_3_CO_12_-MSNs@Ce6@CM peaked at 8 h of intravenous injection. Additionally, MSNs@Fe_3_CO_12_/Ce6@CM exhibited a similar tumor accumulation efficiency to Fe_3_CO_12_-MSNs@Ce6@CM (Supplementary Figure S8). Thus, we used US to irradiate tumors 8 h after administration. Then, we evaluated the antitumor effect of Fe_3_CO_12_-MSNs@Ce6@CM in the presence of US in combination with immune checkpoint blockers. As shown in Figures 4D–F, a single US had a negligible inhibitory effect on tumor progression. Fe_3_CO_12_-MSNs@Ce6@CM slightly delayed the tumor growth, possibly due to the partial release of CO. Notably, Fe_3_CO_12_-MSNs@Ce6@CM plus US showed a remarkable antitumor effect, which displayed higher tumor inhibition rates (69.2%) than MSNs@Fe_3_CO_12_/Ce6@CM plus US (54.4%), further confirming that Fe_3_CO_12_-MSNs@Ce6@CM had excellent SDT sensitization effect owing to its sustained CO release properties. Furthermore, Fe_3_CO_12_-MSNs@Ce6@CM plus US exhibited near-complete tumor eradication when combined with PD-1 antibodies (αPD-1), showing superior efficacy to MSNs@Fe_3_CO_12_/Ce6@CM plus US with αPD-1. Moreover, Fe_3_CO_12_-MSNs@Ce6@CM plus US with αPD-1 resulted in the most CD8^+^ T cells in tumors and the highest release of proinflammatory cytokines, including interleukin-6 (IL-6), interferon-γ (IFN-γ), and tumor necrosis factor-α (TNF-α) (Figures 4G–I; Supplementary Figure S9). These results indicated that Fe_3_CO_12_-MSNs@Ce6@CM-medicated SDT boosted a strong immune activation and thus exerted a synergistic antitumor effect with immune checkpoint blockade treatment to suppress tumor progression.

**FIGURE 5:**
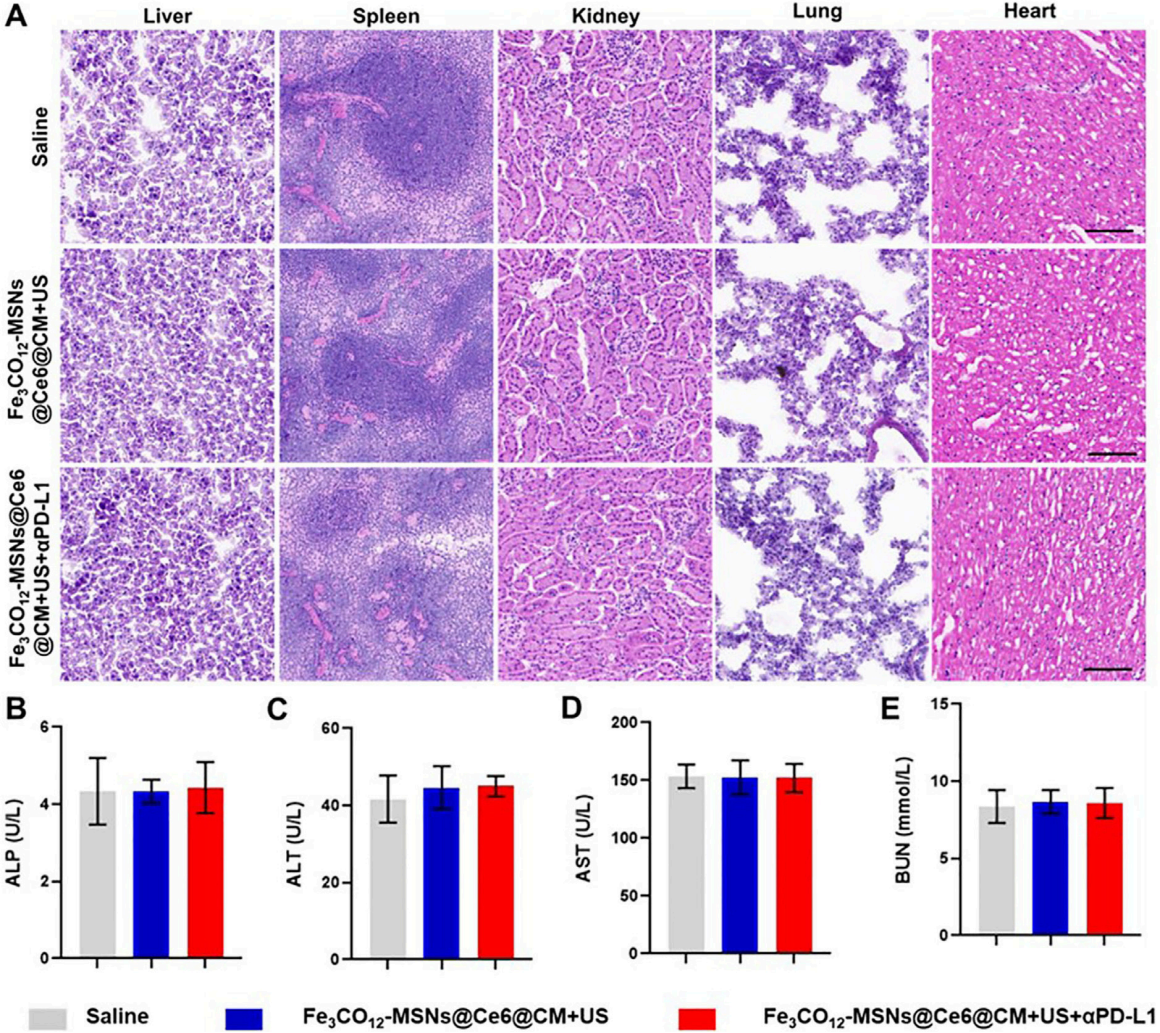
Systemic toxicity of combination therapies. **(A)** H&E staining of the liver, spleen, kidney, lung, and heart after various treatments; scale bar, 100 μm. **(B–E)** Blood biochemical index including ALP **(B)**, AST **(C)**, AST **(D)**, and BUN **(E)** from mice after various treatments; n = 3. Data are presented as the mean ± SD.

Biosafety is an important concern for the application of nanomedicines. Therefore, we investigated the systemic toxicity of Fe_3_CO_12_-MSNs@Ce6@CM-mediated combination treatments by detecting the body weights and serum biochemistry indexes, along with the histology of the major organs. Encouragingly, Fe_3_CO_12_-MSNs@Ce6@CM plus US and Fe_3_CO_12_-MSNs@Ce6@CM plus US with αPD-1 did not lead to a significant decrease in the body weight of mice and obvious changes in serum biochemistry indexes compared with the control groups (Figures 5B–E; Supplementary Figure S10). Additionally, H&E staining indicated that no pathological change was observed in major organs, including the liver, spleen, kidney, lung, and heart, of mice after the treatments with Fe_3_CO_12_-MSNs@Ce6@CM plus US or Fe_3_CO_12_-MSNs@Ce6@CM plus US with αPD-1 (Figure 5A). These results confirmed that Fe_3_CO_12_-MSNs@Ce6@CM-mediated combination therapies had low side effects.

## 4 Conclusion

In summary, we fabricated US-responsive Fe_3_CO_12_-MSNs to load sonosensitizer Ce6 for enhanced SDT of breast cancer. Fe_3_CO_12_-MSNs showed high Ce6-loading ability, ultrastability, and sustained CO release with the stimulus of US irradiation and H_2_O_2_ addition, thus decreasing unwanted CO leakage under physiological conditions and prolonging the therapeutic window in tumors, which effectively sensitized SDT. After coating with cancer cell membranes, Fe_3_CO_12_-MSNs@Ce6@CM exhibited increased blood circulation time and enhanced tumor-targeting ability. The *in vitro* and *in vivo* results indicated that Fe_3_CO_12_-MSNs@Ce6@CM-mediated combination treatments of SDT and CO gaseous therapy possessed an excellent antitumor effect and simultaneously elicited an outstanding ICD effect, which was better than that of traditional Fe_3_CO_12_-loaded MSNs. When combined with αPD-1, Fe_3_CO_12_-MSNs@Ce6@CM plus US exerted almost complete elimination of primary 4T1 tumors and suppression of metastatic tumors with low systemic toxicity. Our work offers a promising CO-releasing nanoplatform with organic–inorganic bridged architectures for US-activated cancer combination therapies.

## Data Availability

The original contributions presented in the study are included in the article/Supplementary Material; further inquiries can be directed to the corresponding author.
