# Supplementary material for: Ultrasound-controllable carbon monoxide nano-delivery systems for combined sonodynamic/gaseous therapies
Source: Front Bioeng Biotechnol. 2025 Jun 13;13:1615481. doi: 10.3389/fbioe.2025.1615481 (PMC12202442; doi:10.3389/fbioe.2025.1615481)
Supplement: Supplementary file 1 [file Supplementaryfile1.docx]

Supplementary Material

**Ultrasound-controllable Carbon Monoxide Nano-delivery Systems for Combined Sonodynamic/gaseous Therapies**

**Chong Feng^1^, Shuang Song^1^, Xiaoyu Zhang^2^, Jing Wang^3^, Qingxin Meng^4^, Tao Wang^4,^ ***

^1^ Ultrasound Department of Hong Qi Hospital of Mudanjiang Medical University, Mudanjiang 157011, China.

^2^ Health Management Center, Mudanjiang First People's Hospital, Mudanjiang 157011, China.

^3^ Department of Biology, School of Basic Medical Sciences, Mudanjiang Medical University, Mudanjiang 157011, China.

^4^ Ultrasound Department of Second Hospital of Mudanjiang Medical University, Mudanjiang 157009, China.

* Correspondence:
Corresponding Author
Echowong8921@126.com

# Supplementary Materials

Treaethyl orthosilicate (TEOS), (3-Mercaptopropyl)trimethoxysilane (MPTES), cetyltrimethylammonium bromide (CTAB), triethanolamine (TEA), 3-aminopropyltriethoxysilane (APTES) were purchased from Sigma-Aldrich Co. (St Louis, MO, USA).Dodecacarbonyltriiron (Fe_3_(CO)_12_, 96%) were purchased from Xin Ding Pengfei Technology Development Co., Ltd (Beijing, China). The CO probe (FL-CO-1), fluorescein isothiocyanate isomer (FITC), ROS assay kit, 4',6-diamidino-2-phenylindole (DAPI) was purchased from Xian Ruixi Biological Technology Co., Ltd (Xian, China). Anti-CD3-PerCP/cy5.5, and anti-CD8a-APC were phurcased from BioLegend, Inc. Rabbit anti-HMGB1 antibody (ab227168) and rabbit anti-calreticulin antibody (ab223614) were obtained from Abcam (MA, USA). Cell Counting Kit-8 (CCK-8) was purchased from Dalian Meilun Biotechnology Corporation (Dalian, China).

# Supplementary Figures

**
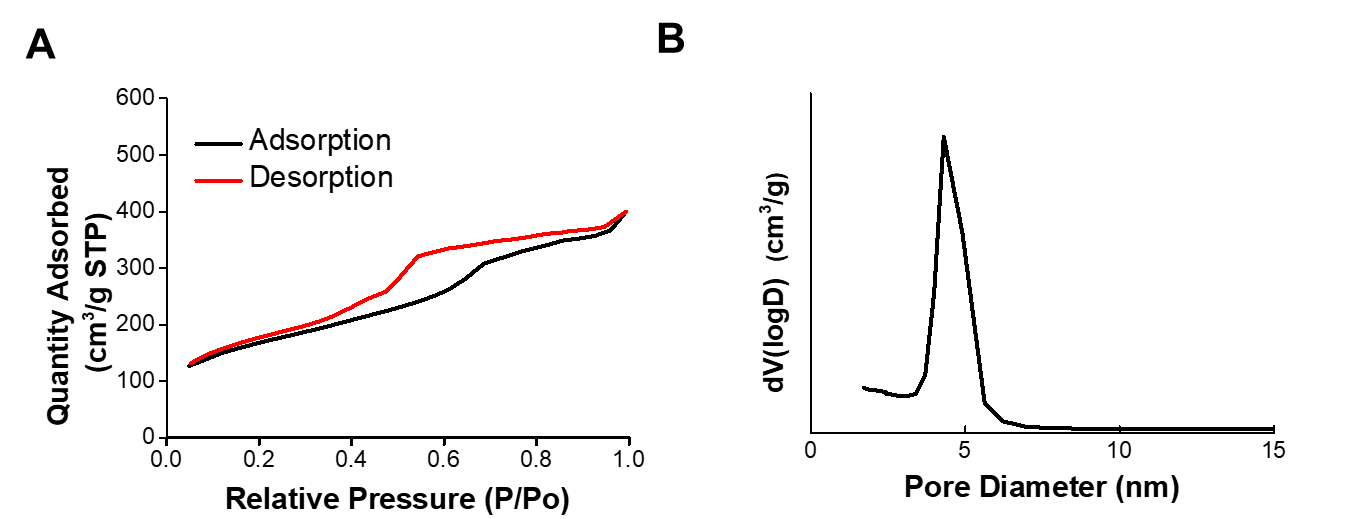
**

**Supplementary Figure 1.** (A) Nitrogen adsorption-desorption isotherms of Fe_3_CO_12_-MSNs. (B) Pore size distribution of Fe_3_CO_12_-MSNs.


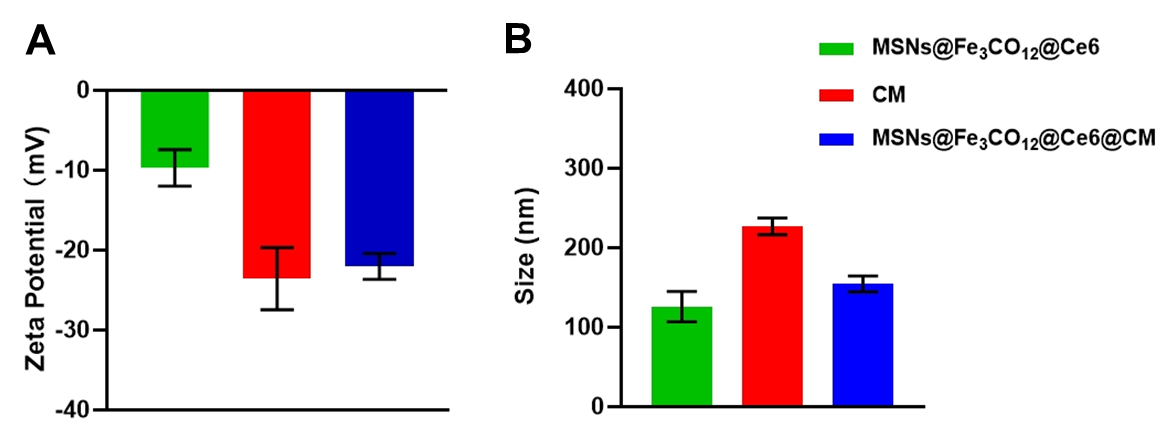


**Supplementary Figure2.** Characterization of MSNs@Fe_3_CO_12_/Ce6@CM. (A) Surface zeta potential of MSNs@Fe_3_CO_12_/Ce6, CM vesicles and MSNs@Fe_3_CO_12_/Ce6@CM, n =3. (B) Size distribution of MSNs@Fe_3_CO_12_/Ce6, CM vesicles and MSNs@Fe_3_CO_12_/Ce6@CM, n =3.

*
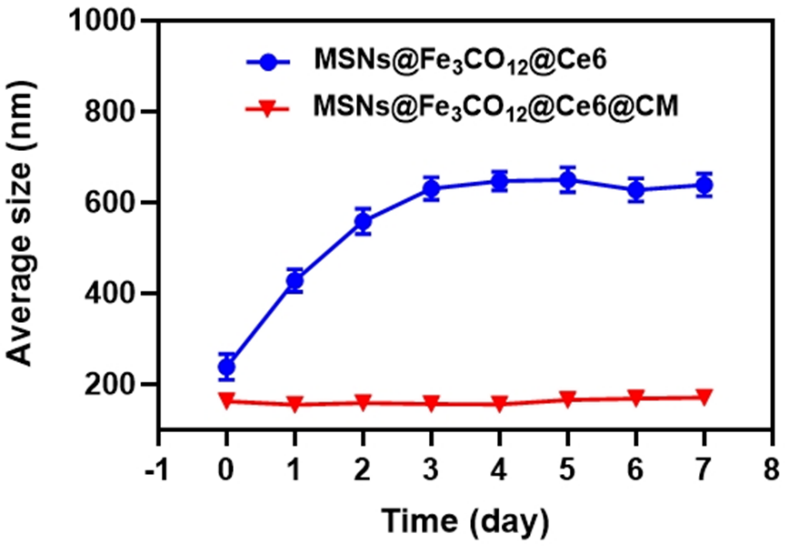
*

**Supplementary Figure 3.** Averaged size of MSNs@Fe_3_CO_12_/Ce6 and MSNs@Fe_3_CO_12_/Ce6@CM after storage in the cell medium, n=3.


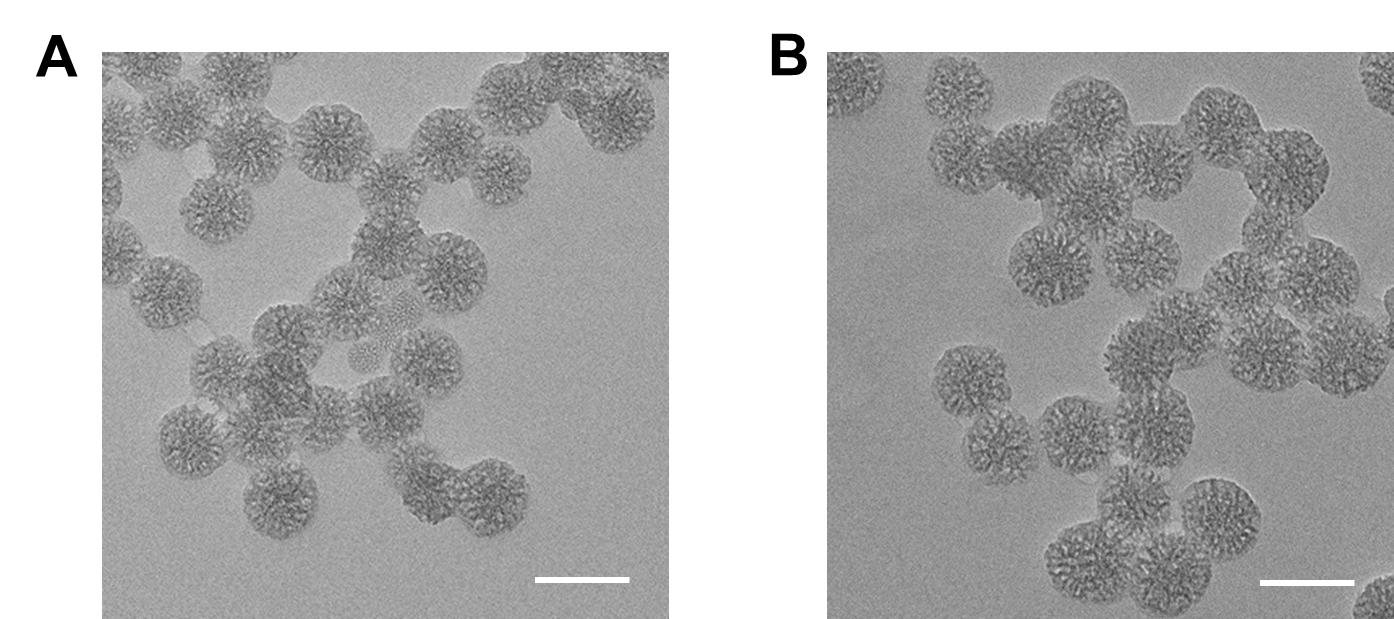


**Supplementary Figure 4.** (A) TEM image of MSNs, the scale bar =100 nm. (B) The morphology of MSNs after treated with 100 μM H_2_O_2_ solution and the US irradiation, the scale bar =100 nm.


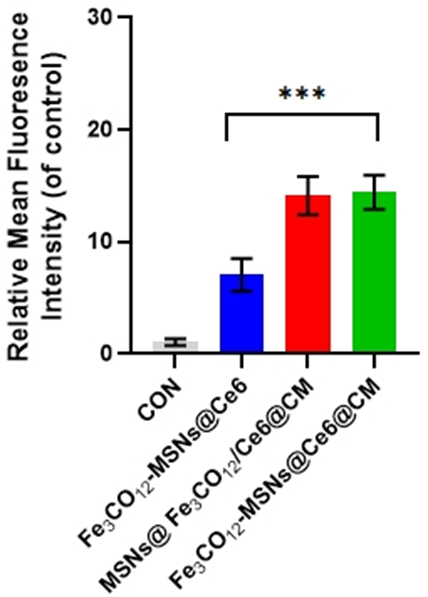


**Supplementary Figure 5.** Quantitative FACS analysis of internalization of Fe_3_CO_12_-MSNs@Ce6@CM, Fe_3_CO_12_-MSNs@Ce6@CM and MSNs@Fe_3_CO_12_/Ce6@CM in 4T1 cells, n = 3, *p < 0.05, **p < 0.01, ***p < 0.001. Data are presented as the mean ± SD.


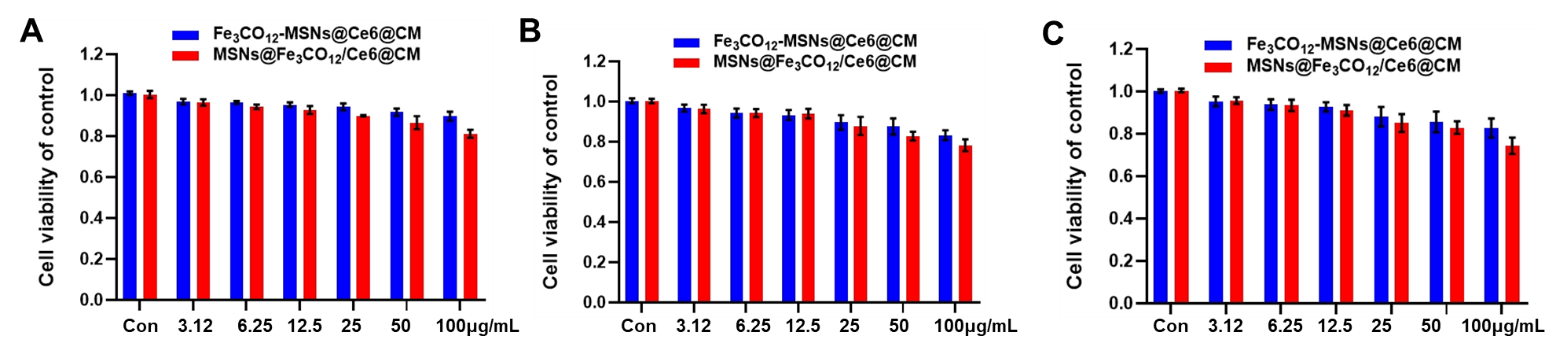


**Supplementary Figure 6.** The cytotoxicity of Fe3CO12-MSNs@Ce6@CM and MSNs@Fe3CO12/Ce6@CM against 4T1 cells (A), MCF-7 cells (B) and HUVECs (C), n =4. Data are presented as the mean ± SD.


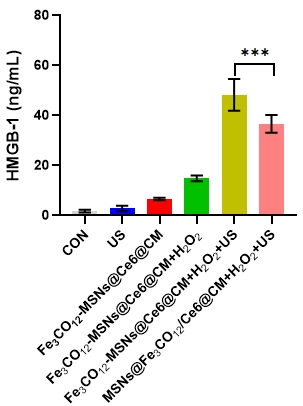


**Supplementary Figure 7.** Secretion of HMGB1 from 4T1 24h after various treatments, n=4, *p < 0.05, **p < 0.01, ***p < 0.001. Data are presented as the mean ± SD.


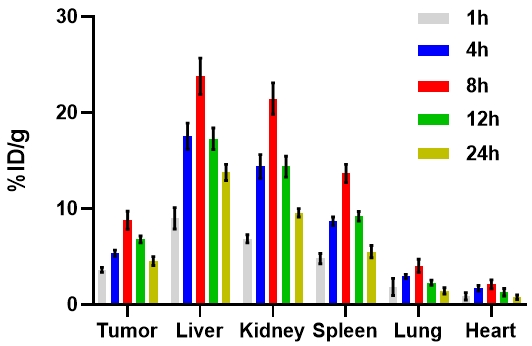


**Supplementary Figure 8.** Biodistribution of MSNs@Fe_3_CO_12_/Ce6@CM after intravenous injection to 4T1 tumor-bearing mice, n =3.


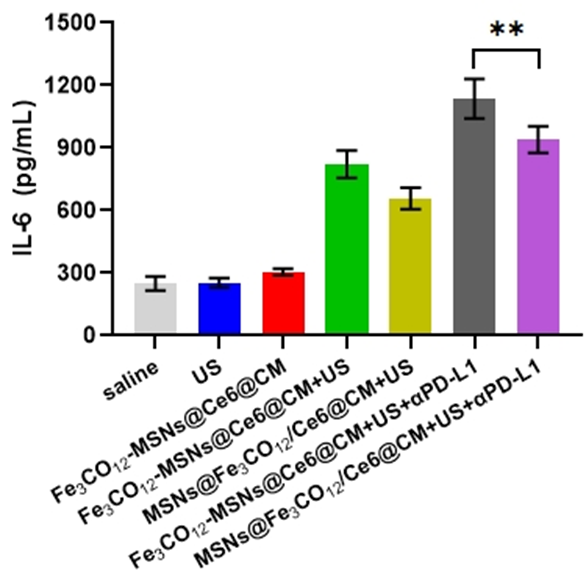


**Supplementary Figure 9.** Secretion of IL-6 various treatments, n=3, *p < 0.05, **p < 0.01, ***p < 0.001. Data are presented as the mean ± SD.


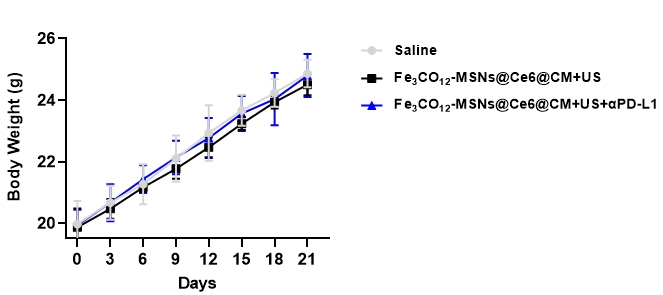


**Supplementary Figure 10.** body weights of mice after various treatments, n =3. Data are presented as the mean ± SD.
